# Histological Findings in the Trabecular Meshwork of a Patient with Atopic Glaucoma

**DOI:** 10.2174/1874364101711010103

**Published:** 2017-05-29

**Authors:** Satoru Kase, Shiki Chin, Teruhiko Hamanaka, Yasuhiro Shinmei, Takeshi Ohguchi, Riki Kijima, Akira Matsuda, Susumu Ishida

**Affiliations:** 1 Department of Ophthalmology, Faculty of Medicine and Graduate School of Medicine, Hokkaido University; N-15, W-7, Kita-ku, Sapporo 060-8638, Japan; 2 Department of Ophthalmology, Teine Keijinkai Hospital; Maeda 1-12, Teine-ku, Sapporo 006-0811, Japan; 3 Department of Ophthalmology, Japan Red Cross Society Medical Centre, Tokyo, Japan; 4 Department of Ophthalmology, Juntendo University Graduate School of Medicine, Tokyo, Japan

**Keywords:** Atopic glaucoma, Trabeculotomy, Histopathology, Intraocular pressure, Postoperative fibrosis, Trabecular meshwork

## Abstract

**Purpose:**

The aim of this study was to report a case of atopic dermatitis showing elevated intraocular pressure (IOP) beyond the baseline levels followed by a modified 360-degree suture trabeculotomy, and to analyze the histological findings in the trabecular meshwork.

**Methods:**

A 40-year-old male suffered from blurred vision in the right eye (OD). He had a medical history of severe atopic dermatitis and intraocular lens implantation OU due to atopic cataract. At the initial presentation, the visual acuity was 0.03, and IOP was 35 mmHg OD. Slit-lamp examination demonstrated corneal epithelial edema OD. Increased IOP was refractory to several topical medications. The patient underwent a modified 360-degree suture trabeculotomy. The visual field defect, however, deteriorated with persistently high IOP. The patient underwent trabeculectomy together with drainage implant surgery. In the outflow routes, although there seemed to be an opening of Schlemm’s canal into the anterior chamber, there was no endothelium of the canal in the region of its opening. The fibrotic changes were conspicuous around Schlemm’s canal.

**Conclusion:**

The histological results indicated that trabeculotomy might not be an appropriate treatment for patients with atopic glaucoma, possibly because of excessive repair to the newly created uveoscleral outflow in addition to the increased postoperative fibrosis in the trabecular meshwork and Schlemm’s canal.

## INTRODUCTION

Nowadays, it is necessary to consider the patient suffering from glaucoma in its entirety. Evaluation of familiar and pathological clinical history is now compulsory and an intrinsic evaluation of the patient is undoubtedly useful; only in this way, it is possible to develop a personalized follow up [1]. Atopic dermatitis can complicate severe ocular disorders such as atopic keratoconjunctivitis [2], atopic cataracts [3] and retinal detachment [4]. Glaucoma associated with atopic dermatitis has been considered one of the adverse effects of glucocorticoids, while the correlation of atopic dermatitis with glaucoma was first predicted in 1960 [5]. However, atopic glaucoma is a novel clinical entity showing increased intraocular pressure (IOP) and glaucomatous optic nerve changes, leading to subsequent severe visual impairment [6]. Atopic glaucoma is likely to be refractory to topical and oral medications to reduce high IOP; therefore, it often requires surgical intervention. However, a useful surgical intervention to control elevated IOP remains unknown. We herein reported a case of atopic dermatitis showing elevated IOP above the baseline levels and treated with a modified 360-degree suture trabeculotomy and then analyzed histological findings in the trabecular meshwork.

## CASE PRESENTATION

According to Takakuwa *et al.* [6], a 40-year-old male can be classified as having atopic glaucoma because 1) this patient has suffered from severe atopic dermatitis, and 2) ophthalmologically, the optic disc revealed glaucomatous cupping having a vertical cup ratio of >0.7 with compatible visual field loss and increased IOP measuring over 21 mm Hg.

In the initial interview in October 2012, visual acuities were 0.03 OD and 1.5 OS. IOP was 35 mmHg OD and 14 mmHg OS. Slit-lamp examination demonstrated corneal epithelial edema with intraocular lens (IOL) implantation OD. Gonioscopy revealed an open angle OD. The fundus was invisible OD. The left eye showed nothing of note except for IOL implantation. The Humphrey visual field revealed mild depression OD Fig. (**1**). Although he received several topical anti-glaucoma agents and oral acetazolamide, the IOP of his right eye remained high, measuring around 35 mmHg. The patient underwent a modified 360-degree suture trabeculotomy in June 2013, as reported previously [7]. Briefly, after making a scleral flap, a corneal side port incision was created opposite to the scleral flap to cleave the Schlemm’s canal using 5-0 nylon suture. The visual field defect, however, deteriorated with persistently high IOP Fig. (**1**). While IOP increased to more than 40 mmHg, the patient underwent a trabeculectomy together with drainage implant surgery in December 2013. The IOP remained favorable in June 2014. In brief, a trabeculectomy was additionally performed at the side of the tube to prevent intraocular hypertension early after implantation of Baerveldt^®^ glaucoma implant to the superior-temporal site of the anterior chamber. We conducted a histopathological investigation using trabeculectomy specimens which included the area of the previous trabeculotomy, in order to elucidate the mechanisms underlying the increased IOP beyond the baseline level.

The visual field reveals mild depression in the right eye at the initial presentation in October 2012. Marked progression of the visual field defect is noted one year later in October 2013.

Schlemm’s canal seems to open into the anterior chamber (A, arrowhead). However, endothelium of the canal is absent in the supposed region of the canal (A), and there is almost no positive reaction to thrombomodulin (B) and CD34 (C) immunohistochemical staining, except for the small remnant of the canal (B, arrows).

The TEM image is of the boxed area (D). The star and dotted arrow in D indicate fibrotic changes around the supposed region of the canal and the track of trabeculotomy, respectively. TEM reveals fusion of the trabecular beams (E, stars), with the collection of 30-nm materials (F). Figure F is a magnification of the boxed area in E.

### HISTOLOGICAL FINDINGS

**Fig. (2):**
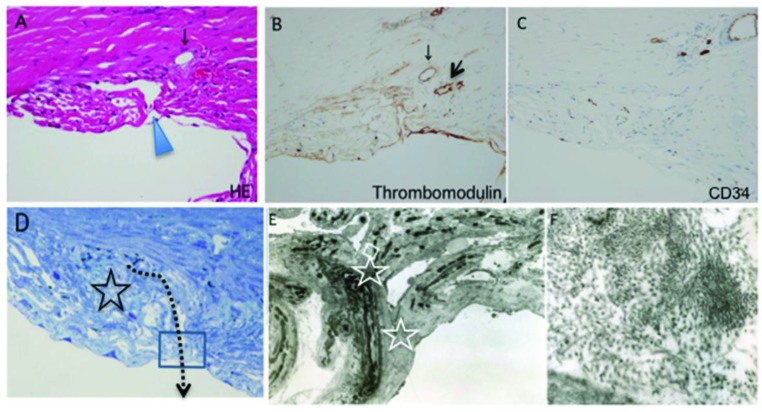
Hematoxylin & eosin staining (A), immunohistochemistry for thrombomodulin (B) and CD34 (C), toluidine blue staining (D), and transmission electron microscopy (TEM) (E, F) in trabecular beam tissue obtained during trabeculectomy.

In the outflow routes, there seemed to be an opening of Schlemm’s canal into the anterior chamber (Fig. (**2A**), arrowhead). However, in the region of the opening, there was no endothelium of Schlemm’s canal Fig. (**2A**) and no positive reaction to thrombomodulin Fig. (**2B**) and CD34 Fig. (**2C**) immunohistchemical staining. The fibrotic changes were conspicuous around Schlemm’s canal (Fig. (**2D**), star). In the area of the trabeculotomy (Fig. (**2D**), dotted arrow), transmission electron microscope (TEM in the box of Fig. **2D**) findings revealed trabecular beams fused to each other (Fig. (**2E**), stars). A collection of electron-dense fibers with a diameter of ca. 30 nm was noted in the corneoscleral meshwork Fig. (**2F**).

## DISCUSSION

We analyzed the histopathology of the trabecular meshwork in a patient with atopic dermatitis and glaucoma, who underwent a modified 360-degree suture trabeculotomy. Trabecular tissue obtained during the trabeculectomy revealed that the outflow routes around Schlemm’s canal had been obliterated by fibrotic changes. Thirty-nanometer electron-dense fibers were accumulated in the corneoscleral meshwork, which is consistent with findings observed on atopic glaucoma [6]. Moreover, histological findings also showed no positive reaction for thrombomodulin and CD34 Figs. (**2B**, **C**), which serves as a marker for endothelium of the canal on immunohistochemical staining [3]. These results suggest that the lumen of Schlemm’s canal was not opened into the anterior chamber because endothelium of the canal was not observed.

We previously reported that a modified 360-degree suture trabeculotomy is a useful method to control postoperative IOP in patients with open angle glaucoma [7]. The mechanisms underlying IOP reduction following a trabeculotomy may be associated with enhanced uveoscleral outflow because the opening of Schlemm’s canal into the anterior chamber may not be important for the IOP-lowering effect [8]. Therefore, we initially conducted a modified 360-degree suture trabeculotomy with this patient; however, the IOP increased to more than the baseline before glaucoma surgeries. The histological results indicated that a trabeculotomy might not be an appropriate treatment for patients with atopic glaucoma, possibly because of excessive repair of the newly created uveoscleral outflow in addition to the increased postoperative fibrosis in the trabecular meshwork and Schlemm’s canal.

Based on this report, we recommend that patients with atopic glaucoma should be treated with trabeculectomy if the IOP is not favorably controlled by topical medical therapy. Unless the IOP is controlled following trabeculectomy, a tube implant surgery combined with trabeculectomy should be applied to such patients.

## Figures and Tables

**Fig. (1):**
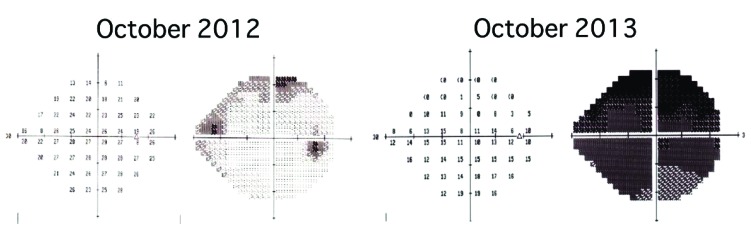
Humphrey visual fields in 0ctober 2012 (left panel) and 2013 (right panel).
